# Silent shapes and shapeless sounds: the robustness of the diminished crossmodal correspondences effect in autism spectrum conditions

**DOI:** 10.1007/s00426-019-01163-9

**Published:** 2019-03-12

**Authors:** Magdalena Ewa Król, Kinga Ferenc

**Affiliations:** Wrocław Faculty of Psychology, SWPS University of Social Sciences and Humanities in Wrocław, Wrocław, Poland

## Abstract

We performed a registered replication of the Oberman and Ramachandran (Soc Neurosci 3(3–4):348–355, 2008) study on the ‘kiki/bouba’ effect in autism spectrum conditions (ASC). The aim of the study was to test the robustness of the diminished crossmodal correspondences effect in autism, but also to verify whether this effect is not an artifact of differences in intelligence. We tested a Polish-speaking sample of 21 participants with ADOS-confirmed autism spectrum conditions (mean age 15.90) and 21 age- (mean age 15.86), sex- and IQ-matched neurotypical control participants. Procedure closely followed the replicated study. Participants’ task was to match five pairs of unfamiliar words and shapes. Matching words and shapes had similar supramodal characteristics that allowed the match. We report partial replication of the diminished ‘kiki/bouba’ effect in individuals with ASC compared to the neurotypical control group. However, we found that nonverbal intelligence also significantly contributed to task performance, but only in participants with autism, suggesting a compensatory role of intelligence. Finally, the effect of autism severity (measured by ADOS classification) was significant—crossmodal correspondences were weaker in individuals with autism, compared to those with autism spectrum diagnosis.

**Electronic supplementary material:**

The online version of this article (10.1007/s00426-019-01163-9) contains supplementary material, which is available to authorized users.

## Introduction

Regardless of their culture, age and native language, people associate rounded shapes with words like ‘bouba’ or ‘maluba’, and spiky shapes with words like ‘kiki’ or ‘takete’ (Bremner et al., 2013; Köhler, 1947; Maurer, Pathman, & Mondloch, 2006). The most likely reasons for the association are that the shape mimics both the waveform of the word and the inflection of the tongue when the word is pronounced (Ramachandran & Hubbard, 2001). The presence of such crossmodal associations shows that the brain is capable of abstracting supramodal similarities between stimuli from different modalities. Furthermore, the brain’s ability to extract such supramodal correspondences is the foundation of metaphorical thinking, i.e., the ability to perceive similarities between distant ideas (the phrase ‘distant ideas’ used here is on its own an example of a cross-modal association) (Marks, 1982; Ramachandran & Hubbard, 2001; Seitz, 2005).

It would thus seem that the presence of cross-modal associations signifies a fundamental property of a typical human brain. Conversely, the absence of this trait would be an equally important sign. Oberman and Ramachandran (2008) showed that crossmodal associations, such as the ‘kiki/bouba’ effect, are less pronounced in people with autism spectrum conditions (ASC). Impaired understanding of metaphors is also one of the hallmarks of autism (Norbury, 2005; Rundblad & Annaz, 2010). Furthermore, people with autism have problems with multisensory integration (Stevenson et al., 2014). Thus, it would seem that the brains of people with autism do not connect and integrate information from different modalities to the same extent as neurotypical brains.

But why does the autistic brain not associate the word ‘kiki’ with jaggedness and ‘bouba’ with roundness? According to Oberman and Ramachandran (2008), decreased multisensory integration may be a signature of dysfunction in the mirror neuron system, which has been shown to be impaired in people with autism (Rizzolatti, Fabbri-Destro, & Cattaneo, 2009). The role of the mirror neuron system is to connect visual and motor representations of the same action. Thus, impairment in the mirror neuron system could lead to difficulties in detecting similarities between visual stimuli and tongue movements, like in the case of the ‘kiki/bouba’ effect. The limitation of this theory is that it explains only cases of sensory-motor similarities. It cannot explain difficulties with multisensory integration and synesthetic metaphors, originating from multisensory similarities.

However, alternatively, the difficulties with multisensory integration could be explained by altered connectivity in the brains of people with autism (Belmonte et al., 2004; Just, Keller, Malave, Kana, & Varma, 2012). Even though the precise nature of atypical connectivity in autism is still disputed (Picci, Gotts, & Scherf, 2016), overall there seems to be a trend towards support for the decreased long-range connectivity (O’Reilly, Lewis, & Elsabbagh, 2017; Vissers, Cohen, & Geurts, 2012). Decreased communication between distant brain regions may impair multisensory integration, behaviorally leading to the decreased occurrence of multimodal correspondences.

Even though this line of research has large explanatory potential, the number of studies on the decreased multimodal correspondences in people with autism is too low to judge the robustness of this effect. Oberman and Ramachandran (2008) performed the first study of this kind, on a sample of native English speaking participants, with mean age 9.7 and no intellectual disability. Ten participants with ASC diagnoses confirmed by ADOS, and 20 neurotypical participants (age-, sex-, and IQ matched to the ASC group) were requested to assign five pairs of words to shapes. The difference between the groups was statistically significant. Neurotypical participants assigned the word to the synesthetically corresponding shape in 88% of cases, but ASC participants only on 56% of cases, which means that their performance was not significantly different from chance. To summarize, the conclusions that can be drawn from the pioneering Oberman and Ramachandran (2008) study are mainly limited by the small sample size.

Occelli, Esposito, Venuti, Arduino and Zampini (2013) performed the second study on this topic. Their sample of native Italian speakers consisted of 37 neurotypical children and adolescents, with a mean age of 13, and 35 children/adolescents with ADOS-confirmed ASC diagnoses, with a mean age of 12. The ASC group was divided into two groups based on IQ tests and clinical interviews: the ‘low-functioning’ group of 20 participants with mean IQ equal to 66 and the ‘high-functioning’ group of 15 participants with mean IQ of 93. The IQ of the neurotypical group was not determined. The groups were not matched in terms of age, sex, and IQ. Participants were requested to assign fourteen pairs of words to shapes, all of which had similar visual and phonetic properties to the usual ‘kiki’ and ‘bouba’ stimuli. The authors found that performance in the task in both the neurotypical and ‘high-functioning’ groups with ASC was significantly different from chance, but in the ‘low-functioning’ group it did not differ from the chance level significantly. There were significant differences in the patterns of responses in all three groups, i.e., between neurotypical and both the ASC groups, as well as between the two ASC groups, with the neurotypical group the most frequently assigning shapes to corresponding words, and the ‘low-functioning’ group the least frequently. To summarize, even though the Occelli et al. (2013) study had a large sample size, the lack of control over IQ, age, and sex limits the conclusions that can be drawn. Given that the ‘high functioning’ ASC group had a slightly below-average IQ (93) and the IQs in the neurotypical group were not determined, the observed pattern of results may be confounded by differences in intelligence. Additionally, given the differences in performance between the ‘low-functioning’ and ‘high-functioning’ groups, the study shows that, apart from autism itself, a comorbid diagnosis of intellectual disability also influences performance in the task. Therefore, intelligence emerges as an important factor and it needs to be carefully controlled in the future studies on the presence of crossmodal correspondences in autism.

The last study published so far on the topic was performed by Gold and Segal (2017) on a Hebrew-speaking sample, consisting of 20 adolescents and young adults with ASC diagnoses (mean age 18.16), and 20 age- and sex-matched neurotypical adolescents (mean age 18.13). The task and stimuli were similar to the previous two studies, and five pairs of stimuli were used in the task. Additionally, the assessment included Raven Progressive Matrices as the measure of nonverbal intelligence and the Autism Spectrum Quotient (Baron-Cohen, Wheelwright, Skinner, Martin, & Clubley, 2001) questionnaire filled by the caregivers as a measure of autistic traits. The authors found significant differences between the groups on all measures, which included both lower intelligence and weaker crossmodal correspondences in the ASC group. Moreover, performance in the ‘kiki/bouba’ task did not correlate with any of the psychometric measures, with the exception of the AQ score, which reached statistical significance only in the ASC group (higher AQ scores correlated with weaker crossmodal correspondences). However, the AQ test is not intended as a diagnostic test of autism or its severity (Baron-Cohen et al., 2001), only as a measure of the degree of autistic traits in adults of normal intelligence. Both autism diagnosis and autism severity are usually measured using the ADOS (Lord et al., 2012), which is the golden standard in the diagnosis of autism, both for clinical and research purposes, but which was not used in this study. Finally, the groups were not matched in terms of intelligence and, in fact, there was a large and significant difference in IQ between the groups. Intelligence was controlled for only by means of statistical analyses—the authors conclude that intelligence was an unlikely contributor given the lack of statistically significant correlation between task performance and IQ in either of the groups. This may be misleading, because performing the correlation analyses on two groups that differed in terms of intelligence decreased the range of intelligence in each group. It is possible that the within-group differences in IQ were too small compared to the level of variance, occluding the real relationship between IQ and performance. Therefore, the contribution of intelligence to the difference between autistic and non-autistic participants in performance in the ‘kiki/bouba’ task remains indeterminate.

The purpose of this study was to replicate the Oberman and Ramachandran (2008) experiment, to verify whether the difference between the ASC and neurotypical participants in the ‘kiki/bouba’ task would be present. Additionally, we wanted to determine the role of intelligence in this relationship. Given that this study is a replication, we pre-registered the study protocol on OSF (registration can be accessed here: https://osf.io/44hg6/, 10.17605/OSF.IO/44HG6) on 14th February 2017. The goal of preregistration is to increase the validity and replicability of research findings, by committing to a protocol of data collection and analysis before the data are actually collected (Nosek et al., 2015; Nosek & Lakens, 2014; Simmons, Nelson, & Simonsohn, 2011; Wagenmakers, Wetzels, Borsboom, van der Maas, & Kievit, 2012). This eliminates the option of changing the methods of data collection, processing and analysis until a significant result is found. Wagenmakers et al. (2012) propose to label only preregistered analyses as ‘confirmatory’, while all other analyses, while valuable and encouraged, may only be labeled as ‘exploratory’, which changes the reliability of the conclusions based on these analyses. For this reason, all analyses in this study are classified as confirmatory (which means they were included in the pre-registration) or exploratory (which means that they were not included in the pre-registration). In the pre-registration, we have formulated the following research questions: (1) will the Oberman and Ramachandran study replicate? That is, will the ‘kiki/bouba’ effect be present to a smaller extent in the patterns of responses in the ASC group, compared to the control group? (2) Is the presence of the ‘kiki/bouba’ effect dependent on the intelligence in either of the groups? (3) Is the ‘kiki/bouba’ effect related to the severity of autism symptomatology, as measured by ADOS? In other words, would individuals with more severe autistic symptoms, that were assigned the diagnosis of autism according to the ADOS scoring algorithm, have more difficulties with the crossmodal correspondences task compared to individuals that were assigned the less severe autistic spectrum classification? Finally, in the preregistration we hypothesized a significant relationship between the ‘kiki/bouba’ effect and the degree of autistic traits, as measured by the AQ test.

However, even though we obtained AQ scores for our participants, we decided not to include them in the analysis. This is because in some cases the participants themselves completed the AQ measure while in other cases the AQ was completed by the participants parent/guardian (e.g., due to developmental difficulties). Therefore, it is not possible to meaningfully compare AQ scores calculated from responses of the individuals themselves and those obtained from the parent describing the individual, as previous studies have shown that AQ scores obtained from the parents are higher (Baron-Cohen et al., 2001; Wakabayashi, Baron-Cohen, Wheelwright, & Tojo, 2006).

## Method

### Participants

The sample consisted of 42 participants: 21 neurotypical participants with no history of ASC diagnoses or any other developmental disorders (four females, mean age 15.86, SD 2.72) and 21 participants who have been diagnosed with autism spectrum conditions (four females, mean age 15.90, SD 4.39). The study was approved by the faculty ethics committee, in accordance with the 2013 version of Declaration of Helsinki, which stipulates that every research study involving human participants must be registered in a publicly accessible database before recruitment begins. Written, informed consent to take part in the study was obtained from all adult participants, or from their parents or caregivers, in case of underage participants. All participants had given oral consent.

All participants with ASC were independently diagnosed by a psychiatrist, based on the criteria outlined in ICD-10 (World Health Organization, 1992). Fifteen were clinically diagnosed with Asperger’s syndrome and six with autism. All diagnoses were confirmed during the study using the Autism Diagnostic Observation Schedule 2 (ADOS-2; Lord et al., 2012), which is a standardized, validated instrument for assessment of autism spectrum conditions. One participant was excluded due to the lack of an earlier ASC diagnosis done by an independent clinician. Of the remaining sample of 21 participants, 8 met the less stringent ADOS cutoff for autism spectrum and 13 met the criteria for autism. The minimum number of points for autism spectrum diagnosis is 7 in both modules 3 and 4, while for the classification of autism, the minimum is 9 in module 3 and 10 in module 4. The exclusion criteria for all participants were: vision uncorrected to normal, neurological and genetic conditions, intellectual disability, defined as IQ below 70 in Wechsler test. However, the lowest IQ score in the study was 85, so significantly above the minimum criterion. Depending on the participant’s age, IQ was determined by either Wechsler Intelligence Scale for Children—Revised (WISC-R, Wechsler, 1974, for participants younger than 17) or Wechsler Adult Intelligence Scale (WAIS-R, Wechsler, 1983, for participants aged 17 or above). All participants were native Polish speakers. The groups were matched in terms of age, sex and IQ [full, performance IQ (PIQ) and verbal IQ (VIQ) (Table 1)].

**Table 1:** Participants’ characteristics

	ASC group (*N* = 21)		TD group (*N* = 21)		*p*
	Mean	(SD)	Mean	(SD)	
Age	15.90	4.39	15.86	2.72	0.35^a^
Full scale IQ	107.71	15.95	110.19	8.90	0.54^b^
Verbal IQ	109.67	17.40	107.29	9.28	0.58^b^
Performance IQ	104.19	17.30	112.19	11.94	0.09^b^

The last column (*p*) presents *p* values for the between-group differences

^a^Mann–Whitney test

^b^*t* Test

### Stimuli

Method and stimuli closely followed the protocol used by Oberman and Ramachandran (2008).

**Fig. 1:**
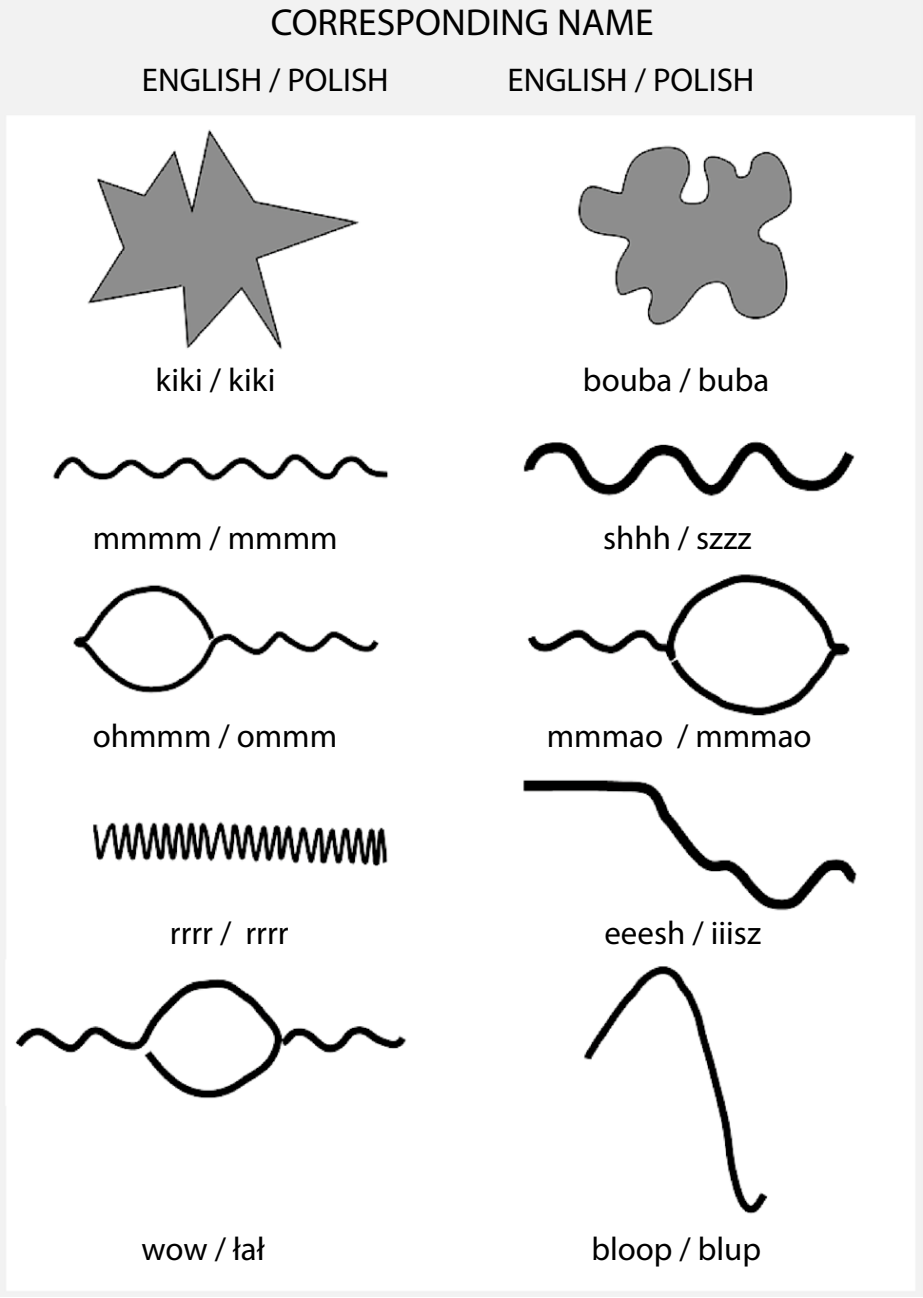
Stimuli used in the study, based on Oberman and Ramachandran (2008). Below each shape, the first word is the name used in the original study and the latter word is the Polish version used in our study

The stimuli were five pairs of nonsense shapes and five pairs of nonsense words. The stimuli were prepared in such a way that shapes and words were corresponding to each other based on the auditory and visual forms (Fig. 1).

Visual stimuli were prepared as vector images, based on the stimuli used by Oberman and Ramachandran (2008). Shapes were printed in pairs, with two versions of each stimuli pair, positioned in a different order (to the left or to the right). Words were pronounced by the experimenter in a way as similar to the English original version as possible, but with Polish accent and pronunciation of phonemes (for example, rolled Polish ‘r’).

### Procedure

The experiment consisted of five trials, with each stimuli pair presented to each participant once, in randomized order. The instruction from the Oberman and Ramachandran (2008) study: “In Martian language one of these shapes is a [word x] while the other is a [word y], which one is which?” was translated to Polish: ‘W języku marsjańskim jeden z tych kształtów nazywa się [x], a drugi [y], który jest który?’, and then presented verbally by the experimenter. Words were presented in randomized order. Participants responded verbally and by pointing to the shape, their responses were recorded by the experimenter.

## Results

### Chi-square tests (confirmatory analysis)

The distribution of all responses is presented in Table 2.

**Table 2:** Number of participants who gave correct responses in each group for each stimuli pair

	Kiki/Bouba	Mmmm/Shhh	Ohmmm/Mmmao	Eeesh/Rrrr	Wow/Bloop
ASC (*n* = 21)	17	6	11	15	10
TD (*n* = 21)	20	4	15	20	12

Firstly, we tested whether the crossmodal correspondence effect was present in each of the five pairs of stimuli in the control group. We performed five Chi-square tests to find out whether the responses in the TD group differed from the chance level. Responses for the ‘wow/bloop’ pair did not significantly differ from chance (*χ*^2^ = 0.43, *p* = 0.51). Additionally, responses for the ‘mmmm/shhh’ differed significantly from chance (*χ*^2^ = 8.05, *p* = 0.01), but in the opposite direction to the pattern assumed by Oberman and Ramachandran (2008). This could be due to linguistic or cultural differences between Polish and English versions of the stimuli. For this reason, we eliminated these two stimuli pairs from further analyses, as specified in the pre-registration of the study. Responses to all other stimuli pairs in the control group displayed a pattern consistent with the presence of the crossmodal correspondence effect (for simplicity, we refer to such responses as correct), with the following Chi-square values: ‘kiki/bouba’—*χ*^2^ = 17.19, *p* < 0.001, ‘ohmmm/mmmao’—*χ*^2^ = 3.86, *p* = 0.05, ‘rrrr/eeesh’—*χ*^2^ = 17.19, *p* < 0.001.

Next, we performed a three-way Chi-square test to find out whether the frequency of correct responses was different in the ASC and TD groups. Factors included: ASC diagnosis (ASC vs TD), response (correct vs incorrect) and stimuli pair (‘kiki/bouba’, ‘ohmmm/mmmao’, ‘rrrr/eeesh’). We found a statistically significant difference between the groups for the ‘rrrr/eeesh’ pair, *χ*^2^(1) = 4.29, *p* = 0.04, but the differences for the ‘kiki/bouba’ pair (*χ*^2^(1) = 2.04, *p* = 0.15) and ‘ohmmm/mmmao’ pair (*χ*^2^(1) = 1.62, *p* = 0.20) did not reach statistical significance. However, the main effect of the three-way Chi square test was significant, which means that overall, participants with ASC diagnoses responded correctly less often than TD participants, *χ*^2^(1) = 6.61, *p* = 0.01.

Given that responses for the ‘mmmm/shhh’ differed significantly from chance [but in the opposite direction to the Oberman and Ramachandran (2008) study], it could suggest the presence of a reverse crossmodal correspondence in the Polish population. For this reason, we performed an analogous Chi-square test with four, instead of three pairs of stimuli (excluding only the ‘wow/bloop’ pair), reversing the scores for the ‘mmmm/shhh’ pair. There was no significant difference between the groups for the ‘mmmm/shhh’ pair (*χ*^2^(1) = 0.56, *p* = 0.47), but the main effect was still significant (*χ*^2^(1) = 6.67, *p* = 0.01).

We have also performed an analogous Chi-square test with all five pairs of stimuli (including the two pairs we excluded), to perform direct replication of the Oberman and Ramachandran (2008) study. For both the ‘mmmm/shhh’ pair (*χ*^2^(1) = 0.53, *p* = 0.47) and the ‘wow/bloop’ pair (*χ*^2^(1) = 0.38, *p* = 0.54), there were no significant between-group differences. Finally, the main effect was not significant, i.e., there was no significant difference in task performance between the ASC and control participants, *χ*^2^(1) = 2.91, *p* = 0.09.

### Linear regression (exploratory analysis)

The statistically significant difference between the ASC and TD groups in their patterns of responding could be a result confounding factors, such as differences in intelligence. Thus, the question is whether presence of the crossmodal correspondence effect is dependent on intelligence and if it is, whether the difference between the ASC and TD groups can be attributed to differences in intelligence.

To answer this question, initially, we tried mixed-effects modeling, with single response (correct or incorrect) as the outcome variable, and with ASC diagnosis, age and IQ as fixed effects and stimuli pair as a random effect. However, most likely due to a small sample size, it was not possible to achieve convergence for the model. This is a symptom of overparameterization, i.e., fitting more parameters than the data allow and the recommendation in such cases is to reduce the random effects structure (Bates, Kliegl, Vasishth, & Baayen, 2015; Matuschek, Kliegl, Vasishth, Baayen, & Bates, 2017). In this case, given that we had only one random effect variable (stimuli pair), we decided to run a hierarchical linear regression. We aggregated the responses for each participant and, thus, the outcome variable in the model was the number of correct responses in the task. Preliminary bivariate correlations indicated that age (*r*_s_ = 0.23, *p* = 0.15) and VIQ (*r*_s_ = 0.06, *p* = 0.73) were not significantly related to the outcome variable; for this reason, we did not include them in the model. We did not include sex in the model because the number of women in the sample was too low to achieve a meaningful result. We included PIQ (block 1) and ASC diagnosis (block 2) as predictors. The reason why we decided to choose PIQ instead of full-scale IQ is twofold. Firstly, even though there were no statistically significant differences in full scale, verbal and performance IQ between the groups, the difference in PIQ between the groups is actually quite large and, thus, could have a confounding effect. Secondly, PIQ correlated much more strongly with the outcome variable (*r*_s_ = 0.38, *p* = 0.01) than full-scale IQ (*r*_s_ = 0.28, *p* = 0.08). Results of the regression analysis are presented in Table 3. The regression equation was significant for both models, in Step 1: *F*(1,40) = 8.36, *p* = 0.01, with *R*^2^ = 0.17, and in Step 2: *F*(2,39) = 7.46, *p* < 0.01, with *R*^2^ = 0.28. We found that both ASC diagnosis and PIQ significantly predicted accuracy of responses in the task. Specifically, higher performance IQ and absence of ASC diagnosis both predicted higher scores in the crossmodal correspondences task.

**Table 3:** Results of hierarchical linear regression analysis predicting accuracy of responses in the task (*n* = 42)

Variable	*B*	SE (*B*)	*β*	*t*	*p*	95% CI (*B*)
Step 1						
Intercept	0.31	0.71		0.43	0.67	[− 1.13, 1.74]
PIQ	0.02	0.01	0.42	2.89	0.01*	[0.01, 0.03]
Step 2						
Intercept	0.06	0.68				[− 1.31, 1.43]
PIQ	0.02	0.01	0.33	2.32	0.03*	[0.002, 0.03]
ASC diagnosis	0.45	0.19	0.33	2.37	0.02*	[0.07, 0.84]

*p < 0.05

### Correlations with intelligence in each group (confirmatory analysis)

To test whether the presence of the ‘kiki/bouba’ effect depends on intelligence in either of the groups, we performed correlation analysis between PIQ and task performance separately for each of the two groups (*n* = 21 in each group). In the ASC group, we found a significant correlation between PIQ and crossmodal correspondences task performance, *r*_s_ = 0.54, *p* = 0.01. However, in the neurotypical group, the correlation between the same variables was not significant, *r*_s_ = 0.05, *p* = 0.84.

### Correlations with ASC classification (confirmatory analysis)

We performed correlation analyses on the measures of autism classification in the ASC group (all *n* = 21). Autism classification was derived from ADOS scores that divided ASC participants into two categories of autism severity, in accordance with the scoring algorithm. Participants with a sufficiently large number of points, presenting full clinical picture of autism, were assigned the autism diagnosis, while those with less severe but still clinically significant symptoms were assigned the autism spectrum diagnosis. We found a significant point biserial correlation between accuracy in the task and ADOS classification (autism vs autistic spectrum), (*r*_pb_ = 0.44, *p* = 0.05), indicating more accurate responses in participants with the autistic spectrum classification, compared to the participants with the autism classification. ADOS classification was not significantly correlated either to full-scale IQ (*r*_pb_ = 0.30, *p* = 0.19), nor PIQ (*r*_pb_ = 0.23, *p* = 0.32).

### Correlations with Wechsler subtests (exploratory analysis)

Given that we obtained a signifcant effect of intelligence on task performance, we decided to run exploratory Spearman’s rank-order correlation analysis to test which of the subtests of the Wechsler’s inteligence scale were most highly correlated to performance in the crossmodal correspondences task (Table 4). None of the verbal tasks correlated significantly with the crossmodal correspondences task, but the picture completion task from the performance scale significantly correlated with the ‘kiki/bouba’ task performance (note there was no signifcant difference between the ASC and NT groups in this task: *t*(40) = − 0.69, *p* = 0.50) We can also observe that the correlations for the picture attangement and the digit symbol tasks were close to the significance threshold. Data from the study can be found on OSF: https://osf.io/bvtks/.

**Table 4:** Spearman correlation coefficients between task performance and Wechsler’s IQ scale subtests (*n* = 42)

	Crossmodal correspondences task performance	
	*r*_s_	*p*
Full IQ	0.28	0.08
Verbal IQ	0.06	0.73
Information	0.01	0.96
Vocabulary	0.15	0.36
Arithmetic	0.07	0.66
Comprehension	0.05	0.74
Similarities	− 0.10	0.75
Performance IQ	0.38	0.01*
Picture completion	0.48	0.001*
Picture arrangement	0.29	0.07
Block design	0.14	0.39
Object assembly	0.20	0.20
Digit symbol	0.29	0.06

Participants < 16 were tested with WISC-R and participants > 16 were tested with WAIS-R. We did not include the results of the Digit Span subtest, because it is only available in WAIS-R

*p < 0.05

## Discussion

The purpose of the study was to replicate the study performed by Oberman and Ramachandran (2008) regarding the diminished ‘kiki/bouba’ effect in individuals with autism spectrum conditions. We also wanted to verify whether this effect could be at least partially explained by differences in intelligence. Finally, to bolster the evidence for the connection between autism and diminished crossmodal correspondences, we wanted to investigate the relationship between the ‘kiki/bouba’ effect and autism severity (measured by ADOS classification).

The statistical analysis that included all five pairs of stimuli and as such was a direct replication of the Oberman and Ramachandran (2008) study did not yield a statistically significant result (but there was a trend towards significance). However, in the pre-registration, we have stipulated that stimuli pairs that do not elicit the crossmodal correspondences effect may be excluded from the analysis. There was no crossmodal correspondence effect for one pair of stimuli (‘wow/bloop’) and another pair was related to a response pattern opposite to the one postulated by Oberman and Ramachandran (‘mmmm/shhh’). One possible reason for this discrepancy may be the cultural or linguistic differences between English and Polish. We decided to exclude these two pairs, because inclusion of items that do not elicit the crossmodal correspondence effect increases noise and may occlude real effect. However, given these discrepancies between participants’ responses to some of the stimuli and the overall low number of stimuli pairs, the results have to be interpreted very cautiously. It also has to be noted that direct replication of Oberman and Ramachandran (2008) study failed, despite a larger sample size.

That said, both statistical analyses performed on the remaining three pairs of stimuli, i.e., the confirmatory Chi-square test and the exploratory (not planned in the preregistration) linear regression analysis, confirmed that the patterns of responses in participants with ASC were significantly less influenced by crossmodal correspondences. This brings further modest support for the presence of multisensory integration deficit in ASC proposed first by Oberman and Ramachandran (2008) in English-speaking participants, and next replicated by Occelli et al. (2013) in an Italian-speaking sample, and finally by Gold and Segal (2017) in a Hebrew speaking sample. Here, we replicate the difference in the patterns of responses between Polish-speaking participants diagnosed with autism and neurotypical control group. We have also found a significant effect of performance IQ on responses, which turned out to be confined to the ASC group. Finally, we have found a significant correlation between performance in the task and autism severity (ADOS classification).

### Autism, intelligence, and crossmodal correspondences

So far, the potentially confounding influence of intelligence complicated the interpretation of reported results regarding diminished crossmodal integration in individuals with ASC, compared to the neurotypical individuals. For example, Oberman and Ramachandran (2008) reported that the correlation between task performance and intelligence was not significant, but given their very small sample sizes (10 participants with ASC and 20 participants in the control group) and quite high, albeit not significant, correlation coefficient (*r* = 0.24, *p* = 0.2), this evidence is insufficient to preclude the effect of intelligence. Occelli et al. (2013) did not measure IQ in the control group, so its influence cannot be estimated. However, they found a statistically significant difference in task performance between ASC participants with lower IQ (mean 66) and higher IQ (mean 93), which indicates the possibility that intelligence may be a significant factor. Finally, Gold and Segal (2017) did not match groups in terms of intelligence—in their study, neurotypical groups had significantly higher IQ. However, they checked the within-group relationship between task performance and IQ level and reported the absence of a significant correlation. However, given that the groups differed in terms of IQ, the IQ range in each group was much lower than the overall range, which may be the cause for the lack of significant relationship. Moreover, Hamburg, Startin and Strydom (2017) showed a diminished ‘kiki/bouba’ effect in individuals with Down syndrome with lower cognitive ability scores, but not those with higher scores. This suggests that intelligence affects performance in crossmodal correspondence tasks.

Results of this study also show that nonverbal intelligence is a significant predictor of the crossmodal correspondences task performance. The value of standardized coefficients for nonverbal intelligence (PIQ) and ASC diagnosis is actually the same in our study (*β* = 0.33), which means that the effect of these two factors on task performance was similar. While the relationship between nonverbal intelligence (PIQ) and task performance was strong, we found no correlation between verbal intelligence (VIQ) and task performance, which is interesting, because the task seems to be at least partially linguistic in nature. Thus, whatever is the nature of the mechanism behind diminished crossmodal correspondences in ASC, it is probably not language related. Finally, the correlation between crossmodal correspondences task and performance IQ was present only in the ASC group, with no such relationship present in the neurotypical group.

Our results show that the effect of diminished crossmodal correspondences in autism stands, even after accounting for the effect of intelligence. However, the question remains regarding the nature of the effect of intelligence. It could be argued that the effect may be trivial, given that learning disability has a nonspecific detrimental effect on most cognitive tasks. Occelli et al. (2013) and Hamburg et al. (2017) reported decreased ‘kiki/bouba’ effect in participants with intellectual disability, while Oberman and Ramachandran (2008) and Gold and Segal (2017), who tested participants within the normal range of intelligence, found no such effect. Thus, based on those studies, it could be argued that intelligence contributes to the crossmodal correspondence task performance only in the range of intellectual disability and, hence, its influence may be not specific to the task but related perhaps to poorer understanding of the task. However, the results of our study are not consistent with this interpretation, because we tested participants in the normal range of intellectual functioning. To understand this relationship further, we performed a correlation analysis between each of the subtests of the Wechsler’s IQ scale and crossmodal task performance. There was a significant correlation between task performance and Picture Completion task from the nonverbal scale. Furthermore, there was a trend towards significance for two more nonverbal tasks: Picture Arrangement and Digit Span. All three tasks are related to perceptual organization and perceptiveness, which suggests that those functions are important in the detection of visual-sound correspondences. Finally, we found that the correlation between intelligence and crossmodal correspondences was present only in the ASC group. The level of intelligence was not related to the presence of crossmodal correspondences in the typically developing group with intelligence in the normal range, but higher nonverbal intelligence in autistic individuals was related to stronger crossmodal correspondences. Such pattern of results suggests the presence of a compensatory mechanism. For example, Happé (1995) found that typically developing children reach 50% probability of passing false belief tasks at the age of four, while children with autism reach this level of probability once they achieve on average verbal mental age of 9 years and 2 months. Thus, children with autism may be eventually able to compensate their theory of mind deficits using their intellect. At the same time, in non-autistic children, theory of mind tasks can be performed even by children with learning difficulties, such as children with Down’s syndrome (Baron-Cohen, Leslie, & Frith, 1985).

It seems that a similar pattern is present also in case of crossmodal integration—our results indicate that while typically the ‘kiki/bouba’ effect does not involve substantial intellectual involvement (apart perhaps below the level of intellectual disability, but this may be due to task comprehension problems), in the ASC population higher nonverbal intelligence allows autistic individuals to compensate their crossmodal integration deficit.

### Questions for future research

Thus, the results of this study indicate that the size of the ‘kiki/bouba’ effect is influenced not only by being on the autistic spectrum but also by the level of nonverbal intelligence. Perhaps then, there are other factors that may contribute to multisensory integration. For example, is diminished level of crossmodal correspondence limited to autism spectrum conditions? Is it a signature of brain function typical of autism spectrum conditions, or rather a less specific characteristic representative of a broader range of conditions? There is already some evidence that multisensory integration deficit may be present in dyslexia, for example Drijvers, Dingemanse and Zaadnoordijk (2015) found that the ‘kiki/bouba’ effect is weaker in individuals diagnosed with dyslexia, compared to the control group.

To summarize, given that both ASC diagnosis and nonverbal intelligence contribute to the level of crossmodal correspondences, the question is whether there are any other factors that lead to atypical multisensory integration. Given the preliminary research on diminished multisensory integration in dyslexia, it is possible that decreased ‘kiki/bouba’ effect is not specific to autism. However, given opposing patterns of performance in other tasks putatively related to crossmodal correspondences task (for example lack of impairment in metaphor comprehension in dyslexic individuals), it is possible that in each case the neurocognitive mechanism is different. This lack of clarity shows how little is known about the neurocognitive underpinnings of the ‘kiki/bouba’ effect and its meaning as a signature of atypical brain function.

## Conclusion

To summarize, we performed a pre-registered replication of the Oberman and Ramachandran (2008) study on the ‘kiki/bouba’ effect in autism spectrum conditions. We partially confirmed the diminished crossmodal correspondences effect in Polish-speaking individuals with ASC, compared to the control group. Additionally, unlike previous studies, we found a significant effect of nonverbal intelligence on task performance. Given that intelligence in our sample was in the normal range, it is unlikely that the effect can be explained away as a non-specific effect of intellectual disability (affecting, for example, task comprehension). However, the effect of intelligence was significant only in the ASC group, which suggests that autistic individuals with higher nonverbal intelligence are able to partially compensate the crossmodal integration difficulties. Finally, we found a significant effect of autism severity (measured as ADOS classification), as crossmodal correspondences were weaker in individuals with autism diagnosis, compared to those with autism spectrum disorder diagnosis.

## Electronic supplementary material

Below is the link to the electronic supplementary material.

Supplementary material 1 (PDF 93 KB)

## Data Availability

The datasets generated during and analyzed during the current study are available in the OSF repository, https://osf.io/bvtks/.
