# Supplementary material for: Silent shapes and shapeless sounds: the robustness of the diminished crossmodal correspondences effect in autism spectrum conditions
Source: Psychol Res. 2019 Mar 12;84(6):1507–16. doi: 10.1007/s00426-019-01163-9 (PMC7387372; doi:10.1007/s00426-019-01163-9)
Supplement: Supplementary file 1 — Supplementary material 1 (PDF 93 KB) [file 426_2019_1163_MOESM1_ESM.pdf]

This registration is a frozen, non-editable version of this project (/bvtks/)

Register

Study Information

Title

Authors

Research Questions

Hypotheses

Sampling Plan

Existing Data

Explanation

Data collection procedures

Sample size

Sample size rationale

Stopping rule

Variables

Manipulated

Measured

Indices

Design Plan

Study type

Blinding

Study design

Randomization

Analysis Plan

Statistical models

Transformations

Follow-up analyses

Inference criteria

Data exclusion

Missing data

Exploratory analysis

Scripts

Script

Other

Other

Study Information

Title

Provide the working title of your study. It is helpful if this is the same title that you submit for publication of your final manuscript, but it is not a requirement.

Replication of the Oberman and Ramachandran (2008) study on the multisensory integration deficit in the autism spectrum conditions.

Authors

The author who submits the preregistration is the recipient of the award money and must also be an author of the published manuscript. Additional authors may be added or removed at any time.

Magda

Research Questions

Please list each research question included in this study.

1. Will the Oberman and Ramachandran (henceforth O and R study) study replicate? I.e. whether autistic participants will exhibit the kiki/bouba effect with the classic kiki/bouba stimuli, and with four additional pairs of stimuli used in the original study?
2. Is the presence of the kiki/bouba effect dependent on intelligence in either of the studied populations (ASC and controls?)?
3. Is the kiki/bouba effect related to the severity of autism symptomatology, as measured by ADOS?
4. Is the kiki/bouba effect related to the intensity of the autistic traits, as measured by the AQ test? In particular, the imagination subscale?

Hypotheses

For each of the research questions listed in the previous section, provide one or multiple specific and testable hypotheses. Please state if the hypotheses are directional or non-directional. If directional, state the direction. A predicted effect is also appropriate here.

1. Compared to the neurotypical control group, participants with ASC will not display a significant kiki/bouba effect, indicating a deficit in multisensory integration. Firstly, there will be a significant difference between the two groups, indicating a smaller kiki/bouba effect in the ASC group. Secondly, the responses of the ASC groups will not be significant different from random responses, while the responses in the control group will differ significantly from random responses, indicating a presence of the multisenory integration effect.
2. There will be no correlation between overall IQ as measured by the Wechsler test and the presence of the kiki/bouba effect in either of the groups.
3. There will be a correlation between autism severity as measured by ADOS and the presence of the kiki/bouba effect and the kiki/bouba effect, with more severe ASC related to more random responses in the kiki/bouba test.
4. There will be a similar correlation between the AQ test and the kiki/bouba effect, particularly strong for the imagination scale (related to flexibility of thinking and creativity.

Sampling Plan

Existing Data

Preregistration is designed to make clear the distinction between confirmatory tests, specified prior to seeing the data, and exploratory analyses conducted after observing the data. Therefore, creating a research plan in which existing data will be used presents unique challenges. Please select the description that best describes your situation. Please do not hesitate to contact us if you have questions about how to answer this question (prereg@cos.io).

Registration prior to creation of data

Explanation of existing data

If you indicate that you will be using some data that already exist in this study, please describe the steps you have taken to assure that you are unaware of any patterns or summary statistics in the data. This may include an explanation of how access to the data has been limited, who has observed the data, or how you have avoided observing any analysis of the specific data you will use in your study. The purpose of this question is to assure that the line between confirmatory and exploratory analysis is clear.

I have collected data from two participants so far. I have not analyzed these data.

Data collection procedures

Please describe the process by which you will collect your data. If you are using human subjects, this should include the population from which you obtain subjects, recruitment efforts, payment for participation, how subjects will be selected for eligibility from the initial pool (e.g. inclusion and exclusion rules), and your study timeline. For studies that don't include human subjects, include information about how you will collect samples, duration of data gathering efforts, source or location of samples, or batch numbers you will use.

We will recruit ASC participants of both sexes, in the age range of 12-30. The control group will be age and sex matched. The control group will also be matched in terms of the overall IQ as measured by the Wechsler test. We will strive to match both verbal and non-verbal IQ if possible. All ASC participants will have their diagnosis confirmed with ADOS 2. The exclusion criteria will be intellectual disability, epilepsy, vision not corrected to normal, schizophrenia and bipolar disorder (but not depression). All participants will receive voucher cards to thank them for their time and will be reimbursed for inconvenience and expenses. Data collection will run between February and June 2017.

no file selected

Sample size

Describe the sample size of your study. How many units will be analyzed in the study? This could be the number of people, birds, classrooms, plots, interactions, or countries included. If the units are not individuals, then describe the size requirements for each unit. If you are using a clustered or multilevel design, how many units are you collecting at each level of the analysis?

There will be 30 participants in each group (ASC and control). The sample size may changed slightly depending on recruitment - if we are unable to reach 30 ASC participants, it will be a bit lower. However, if recruitment is going well, we will strive to recruit a higher number of participants.

Sample size rationale

This could include a power analysis or an arbitrary constraint such as time, money, or personnel.

The original study had 10 participants, our goal is to at least double that. We will strive to test 30, but given the time and personnel and financial resources needed to recruit and test each participant, it may not be possible.

Stopping rule

If your data collection procedures do not give you full control over your exact sample size, specify how you will decide when to terminate your data collection.

no applicable

Variables

Manipulated variables

Describe all variables you plan to manipulate and the levels or treatment arms of each variable. For observational studies and meta-analyses, simply state that this is not applicable.

Participants will be shown 5 pairs of shapes (copied from the O and R study). They will be instructed that in martian language one of those shapes is called x and the other y, and asked to tell the researcher which name fits each shape. Shapes are printed in pairs on a piece of paper- there are two versions with different orders of the shapes (shape x on the right, and y to the left or vice versa). The order of names given by the researcher is also randomized.

no file selected

Measured variables

Describe each variable that you will measure. This will include outcome measures, as well as any predictors or covariates that you will measure. You do not need to include any variables that you plan on collecting if they are not going to be included in the confirmatory analyses of this study.

Possible covariates: ADOS results, AQ results, Wechsler results. Main measure: participants' responses in the task.

no file selected

Indices

If any measurements are going to be combined into an index (or even a mean), what measures will you use and how will they be combined? Include either a formula or a precise description of your method. If your are using a more complicated statistical method to combine measures (e.g. a factor analysis), you can note that here but describe the exact method in the analysis plan section.

For each participant I will create a mean of responses for the five pairs of stimuli. Each pair will also be analyzed separately to see if the kiki/bouba effect is present for every one of them. If it turns out that one or more stimuli pairs do not elicit the kiki/bouba effect in the neurotypical group, the may be excluded from the analysis of the means.

no file selected

Design Plan

Study type

Please check one of the following statements

Observational Study - Data is collected from study subjects that are not randomly assigned to a treatment. This includes surveys, “natural experiments,” and regression discontinuity designs.

Blinding

Blinding describes who is aware of the experimental manipulations within a study. Mark all that apply.

No blinding is involved in this study.

Study design

Describe your study design. Examples include two-group, factorial, randomized block, and repeated measures. Is it a between (unpaired), within-subject (paired), or mixed design? Describe any counterbalancing required. Typical study designs for observation studies include cohort, cross sectional, and case-control studies.

between groups: ASC vs neurotypical participants, five measurements per participant, the order of the presented shapes randomized, order of the names given by the researcher randomized.

no file selected

Randomization

If you are doing a randomized study, how will you randomize, and at what level?

Randomized order of names and randomized order of shapes

Analysis Plan

Statistical models

What statistical model will you use to test each hypothesis? Please include the type of model (e.g. ANOVA, multiple regression, SEM, etc) and the specification of the model (this includes each variable that will be included as predictors, outcomes, or covariates). Please specify any interactions that will be tested and remember that any test not included here must be noted as an exploratory test in your final article.

chi-square for the main analysis , correlations between test results and iq/ados

no file selected

Transformations

If you plan on transforming, centering, recoding the data, or will require a coding scheme for categorical variables, please describe that process.

no

Follow-up analyses

If not specified previously, will you be conducting any confirmatory analyses to follow up on effects in your statistical model, such as subgroup analyses, pairwise or complex contrasts, or follow-up tests from interactions? Remember that any analyses not specified in this research plan must be noted as exploratory.

not stated at this time

Inference criteria
